# 手术切除巨大胸腔肿瘤1例

**DOI:** 10.3779/j.issn.1009-3419.2010.08.17

**Published:** 2010-08-20

**Authors:** 娟 祁, 义 郭, 军 张, 安光 何

**Affiliations:** 1 110001 沈阳，中国医科大学附属第一医院，中国医科大学肺癌中心 China Medical University Lung Cancer Center, the First Hospital of China Medical University, Shenyang 110001, China; 2 110001 沈阳，中国医科大学附属第一医院肿瘤研究所分子靶向治疗研究室 Department of Molecular Targeted Terapeutics, Cancer Institute, the First Hospital of China Medical University, Shenyang 110001, China; 3 110001 沈阳，中国医科大学附属第一医院胸外科 Department of Toracic Surgery, the First Hospital of China Medical University, Shenyang 110001, China; 4 110001 沈阳，中国医科大学附属第一医院病理科 Department of Pathology, the First Hospital of China Medical University, Shenyang 110001, China

巨大胸腔肿瘤几乎占满一侧胸腔，能被手术彻底切除、治愈者少见；巨大胸膜局限性纤维性肿瘤，属少见（或罕见）胸腔肿瘤；现报告1例胸腔内巨大胸膜局限性纤维性肿瘤：经开胸手术彻底切除肿瘤，患者全身状态迅速恢复正常，随访1年余无复发、身体健康。

## 1 临床资料

患者男性，39岁。胸闷气短半月余，伴低热，无盗汗、咯血，食欲食量下降，体力下降，体重略减轻，丧失劳动能力。查体：一般状态稍差，不发热，面色黄白，结膜略苍白；胸部基本对称，左侧呼吸动度弱，叩诊浊音，呼吸音基本消失。外院胸片及CT检查示：左胸巨大肿瘤几乎占整个左胸腔，左胸腔积液，抽胸水为淡血性，但查瘤细胞及肿瘤系列均为阴性，诊断为胸腔恶性肿瘤、恶性胸膜间皮瘤。于2008年12月23日转入中国医科大学附属第一医院。胸部增强CT：左侧胸膜可见巨大分叶状软组织密度影，边界清晰，其内密度大致均匀，可见片状更低密度区，CT值范围约20 HU-46 HU，横截面大小约16 cm×7 cm；增强后其内可见强化，CT值约62 HU。病变相邻肋骨未见明确骨质破坏，肋间肌未见增厚。左肺下叶受压不张。提示：左侧胸膜占位病变，考虑为胸膜间皮瘤可能性大（图 1）。肺功能检查结果：混合型肺通气功能障碍，小气道功能重度障碍，通气储量百分比为86.1%。

**1:**
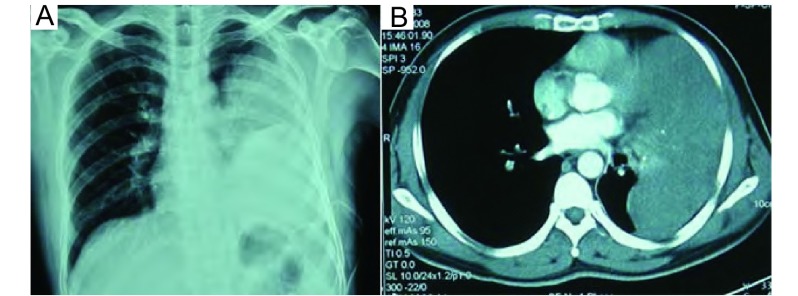
术前X线及CT检查。A：术前胸部X线片；B：术前CT断层扫描均提示巨大胸腔肿瘤，几乎占满整个左胸腔。 Pre-operative chest X-ray and CT. A: Pre-operative chest X-ray; B: Computed-tomography showed the giant tumor occupied almost the whole left thoracic cavity.

于2008年12月31日行胸腔肿瘤穿刺活检，病理诊断：胸膜孤立性纤维性肿瘤（低度恶性）。术前诊断为左胸腔巨大肿瘤、胸膜间皮瘤、胸膜孤立性纤维性肿瘤。

于2009年1月14日在全麻下行左胸巨大“胸膜间皮瘤”切除，左肺上叶部分、左肺下叶部分切除术。术中见肿物与侧胸壁粘连，上达胸膜顶，下方占满肋膈窦，几乎占满整个左胸腔，大小约22 cm×15 cm×7 cm，表面光滑，包膜完整，布满细密毛细血管网；肿物将左肺压向前下方、纵隔面，与左肺上叶尖段S1及左肺下叶背段S6相连，判定其起源于肺脏胸膜，壁胸膜无转移结节。行左肺上叶部分及左肺下叶部分切除术，完整切除肿瘤。瘤体硬韧，实质性，均质，黄白色肉样，中心局部坏死，称重2 kg。

病理检查：切除肿物大小约20 cm×17 cm×6 cm，表面大结节状，包膜完整，表面光滑局部可见少许粘连肺组织，切面色灰白，质韧，局部有囊性改变；光镜所见：瘤组织表面可见完整包膜；瘤细胞长梭形，编织状或漩涡状排列紧密；核卵圆形或梭形，局部可见瘤细胞密集区；间质内见较多胶原成分；免疫组化结果：CD34（+）、CD99（+）、Calretinin（-）、Vimentin（+）、Bcl-2（+）。病理诊断：（胸腔）孤立性纤维性肿瘤（图 2）。

**2:**
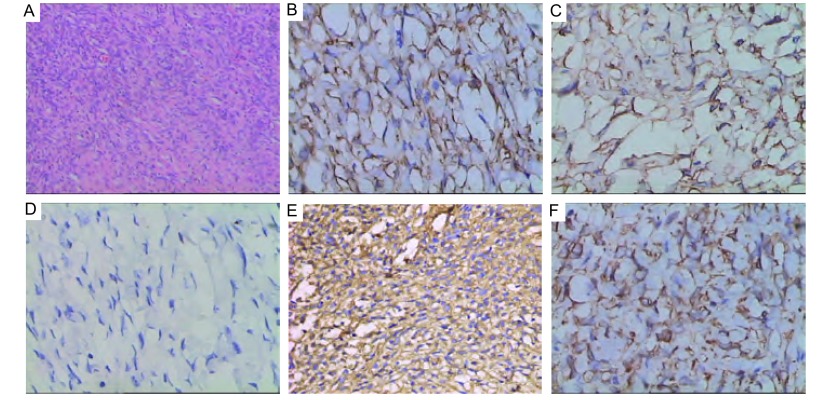
术后病理诊断HE染色（×100）及免疫组化染色结果（×400）；A：HE染色；B：CD34阳性表达；C：CD 99阳性表达；D：Calretinin阴性表达；E：Vimentin阳性表达；F：Bcl-2阳性表达。 Pathological diagnosis with HE staining (×100) and Immunohistochem- ical staining (×400). A: HE staining; B: Positive expression of CD34; C: Positive expression of CD99; D: Negative expres- sion of Calretinin; E: Positive expression of Vimentin; F: Positive expression of Bcl-2.

术后第8天患者治愈出院。术后第1个月、第3个月、第6个月、第12个月定期复诊并行胸CT检查，无肿瘤复发、转移等征象。未进行辅助治疗等。患者面色红润，体重增加，精神状态、身体状态俱佳，完全恢复正常生活状态，并胜任体力劳动。

## 2 讨论

胸膜肿瘤比较少见，多为间皮瘤，一般分为局限性间皮瘤和恶性弥漫性间皮瘤。其中恶性弥漫性间皮瘤较多，多认为其起源于多潜能的间皮细胞，是真性的间皮瘤，与接触石棉有关。而局限性间皮瘤的组织来源既往争议较大，命名也较为混乱，如良性间皮瘤、孤立性纤维性肿瘤、胸膜下纤维瘤等十余种名称^[1]^，现多认为该肿瘤来自间皮下间叶细胞伴有纤维分化倾向，胸膜局限性纤维性肿瘤（localized fibrous tumor of the pleura, LFTP）的命名逐渐得到公认^[2-4]^。

胸膜局限性纤维性肿瘤，多见于中、老年人，女性略多于男性。早期患者一般无症状，多在常规体检或进行胸腔手术时发现^[4]^。随着肿瘤的不断生长，可压迫气管、心肺及交感神经而出现气短、呼吸困难、胸痛、咳嗽等症状、低血糖症状以及肺性骨关节病和杵状指；肺性骨关节病和低血糖症状可在肿瘤切除后迅速缓解，但可随肿瘤的复发而再现^[3]^。本例患者出现胸闷气短症状，主要由于瘤体巨大几乎占据左胸腔，瘤体及胸腔积液压迫心脏及左肺，左肺受压膨胀不全、不张所致。

胸膜局限性纤维性肿瘤多数为良性，少数为低度恶性，多起源于脏层胸膜，也可源于壁层胸膜^[2, 3]^。本例起源于脏层胸膜。胸膜局限性纤维性肿瘤多为孤立局限的肿块，边界清楚，大小不一，平均直径6 cm，大者可达30 cm，多呈分叶状，粘附于脏层胸膜，亦可见于壁层胸膜、叶间裂，可有蒂或无蒂，可单发也可多发^[4, 5]^。良恶性肿瘤在大体上无明显差别，只是恶性者瘤体一般较大，直径多超过10 cm。镜下良性肿瘤主要由纤维母细胞构成，有些区域胶原和网状纤维丰富，细胞呈长梭形，核大卵圆形，核分裂像少见，可见血管成分。恶性者可见多种细胞成分，瘤细胞核呈多形性，且核分裂像多见。与良性胸膜局限性纤维性肿瘤相比，恶性者具有局部侵犯潜能，可累及肺、心包和胸壁，容易复发和转移，预后不良^[1, 2, 6]^。因来源不同，恶性胸膜局限性纤维性肿瘤应与恶性弥漫性间皮瘤应加以区分。

胸膜局限性纤维性肿瘤的X线及CT表现缺乏特异性，易与胸腔包裹性积液、结核性胸膜炎、肺部肿瘤及胸膜转移瘤相混淆；胸腔肿瘤穿刺及开胸探查取得病理活检可确诊。建议术前行胸腔肿瘤穿刺活检，明确病理诊断，对决定手术与否具有重要指导意义。

胸膜局限性纤维性肿瘤的治疗原则是彻底地手术切除，包括瘤体及受累邻近组织。本例为起源于肺组织脏胸膜，楔形切除起源部分肺组织（部分左肺上叶、部分左肺下叶），即将肿瘤一并切除移出胸腔。若瘤体起源于壁胸膜、局部胸壁受累，则需大块胸壁切除以达根治^[6, 7]^。

本例瘤体巨大几乎占满整个左侧胸腔，术前依据影像学资料推测瘤体可能与胸壁严重粘连甚至已侵及胸壁，术前充分估计术中、术后风险，注意术中确切止血、防止胸壁渗血等；防止巨大胸腔瘤体切除后肺复张性肺水肿、纵隔压迫解除后纵隔摆动等引起的严重的心肺并发症。经气管插双腔管麻醉后，轻柔翻身摆右侧卧位，防止突然、暴力翻身导致因瘤体过大、过重突然压迫心脏及对侧肺而诱发心跳骤停或乏氧；因瘤体巨大，术野暴露很重要，本例经左后外侧切口V肋间入胸，断V、VI后肋，瘤体占据整个术野且与胸壁粘连；术中注意动作轻柔、避免挤压瘤体；切除瘤体及部分肺上叶、下叶肺组织后，胀肺前充分吸痰；术中、术后严格控制入液量及输液速度，防止复张性肺水肿等发生。本例手术顺利，术后迅速恢复，第8天即活动如常、治愈出院。术后定期复诊，无肿瘤复发、转移等征象。未进行辅助治疗等。现术后1年多（术后15个月），患者完全恢复正常生活、工作状态。远期疗效有待于长期随访，以决定是否调整治疗策略。
